# Methods for the Assessment of Volume Overload and Congestion in Heart Failure

**DOI:** 10.34067/KID.0000000000000553

**Published:** 2024-08-20

**Authors:** Negiin Pourafshar, Arvin Daneshmand, Ashkan Karimi, Christopher Stuart Wilcox

**Affiliations:** 1 Division of Nephrology, Department of Medicine, Center for Hypertension Research, Georgetown University, Washington, DC; 2 Georgetown University School of Medicine, Washington, DC; 3 Carient Cardiovascular Medicine, Vienna, Virginia

**Keywords:** congestion, volume overload, cardiorenal syndrome, fluid redistribution, echocardiography

## Abstract

Acute decompensated heart failure entails a dysregulation of renal and cardiac function, with fluid volume excess or congestion being a key component. We provide an overview of methods for its assessment in clinical practice. Evaluation of congestion can be achieved using different methods including plasma biomarkers, measurement of blood volume from the volume of distribution of [^131^I]-human serum albumin, sonographic modalities, implantable devices, invasive measurements of volume status including right heart catheterization, and impedance methods. Integration into clinical practice of accessible, cost-effective, and evidence-based modalities for volume assessment will be pivotal in the management of acute decompensated heart failure.

## Introduction

Nephrologists have participated increasingly in the acute and long-term management of patients with heart failure (HF) along with the cardiologists. This overview focuses on methods for assessing fluid overload and congestion, with limited discussion on pathophysiology and treatment because of space constraints. Interested readers are directed to current reviews for further details.^1,2^

The intricate interplay between the kidneys and heart can lead to cardiorenal syndrome, affecting patient outcomes negatively.^3^ Effective decongestion is crucial to prevent mortality and morbidity.^4–6^ Accurate methods for assessing congestion are essential in HF management because inadequate decongestion is associated with higher hospitalization rates and cardiovascular mortality.^6–10^ Residual hypervolemia after acute decompensation is associated with adverse outcomes.^11^

Elevated central venous pressure in HF is associated with both renal dysfunction and unfavorable cardiovascular outcomes.^12–14^ These effects may be compounded by other consequences of venous congestion, including endothelial dysfunction, upregulation of inflammatory cytokines, hepatic dysfunction, and intestinal ischemia.^15,16^ Despite available tools for assessing abnormal fluid volumes during therapy, choosing the best method remains contentious.^17–19^ This review examines various congestion assessment methods, their advantages, and limitations, aiming to address gaps contributing to suboptimal outcomes in HF management.

## Assessment of Congestion

**Figure 1:**
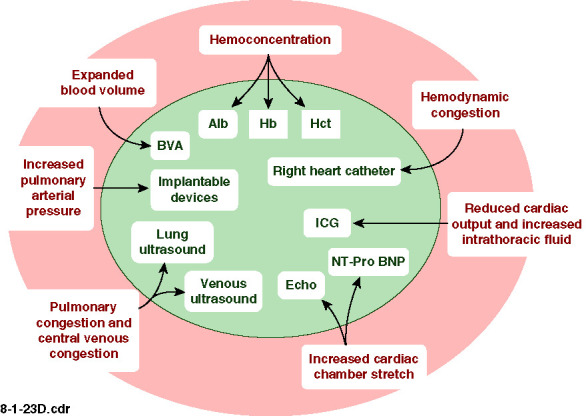
**Methods for assessment of pulmonary and hemodynamic congestion in patients with HF.** Alb, albumin; BVA, blood volume analysis; Hb, hemoglobin; Hct, hematocrit; HF, heart failure; ICG, impedance cardiography; NT-pro BNP, N-terminal pro-brain natriuretic peptide.

Congestion often remains unrecognized until it necessitates hospitalization.^3,20^ Elevated left ventricular filling pressures (*i.e*., hemodynamic congestion) can precede the clinical signs and symptoms of congestion. Indeed, the traditional clinical signs of congestion may not reliably correlate with elevated pulmonary capillary wedge pressure (PCWP).^21^ However, some studies revealed that an elevated jugular venous pulse carries specificity and sensitivity for predicting a future increase in PCWP.^22,23^ This indicates the need for additional means to assess congestion including plasma biomarkers of intracardiac stretch, such as brain natriuretic peptide (BNP) and NT-proBNP; markers of hemoconcentration, such as hematocrit (Hct), hemoglobin (Hb), direct assessment of cardiac contraction, relaxation, and volume by echocardiography; assessment of pulmonary interstitial fluid by lung ultrasound (LUS); direct measurements of blood volume (BV) by the volume of distribution of labeled HSA; indirect measures of cardiac output (CO) and thoracic fluid volume by impedance cardiography (ICG); invasive measurements of central pressures by right heart catheterization (RHC); and implantable devices such as pulmonary artery (PA) sensor/transmitters (Figure 1). In this study, we further discuss these different methods in detail.

## Noninvasive Methods

A summary of noninvasive methods for assessing congestion is highlighted in Table 1.

**Table 1:** Noninvasive methods of evaluating congestion

Method	Pros	Cons
Plasma and blood biomarkers	Noninvasive	Limited specificity (*e.g*., NT-proBNP affected by other conditions)
	Some biomarkers (*e.g*., NT-proBNP) correlate with outcomes	Lack of reliable prediction for mild HF
	Potential for guiding therapy (*e.g*., CA125 in decongestive therapy)	Mixed results in guiding treatment decisions
Echocardiography	Provides direct visualization of heart structure and function	Limited utility in certain conditions (*e.g*., valve disease)
	Can estimate PCWP and evaluate ventricular size/function	E/e′ ratio not always predictive of PCWP
Sonographic assessments	Non-invasive and portable	LUS B lines not specific to ADHF; also seen in other lung pathologies
	Can assess extravascular lung water (LUS) and central BV (IVC). End-expiration IVC diameter >2.1 cm (measured in the supine position in the subcostal long axis, 1–3 cm from the IVC-right atrial junction) and IVC collapsibility index <50% correspond with right atrial pressure >10 mm Hg	Limited evidence supporting use in guiding decongestive therapy
BVA	Quantitative assessment of BV	Requires interpretation in clinical context (comorbidities, medications)
	Helps distinguish intravascular from interstitial fluid volume	Initial measurement needed; ongoing changes in red cell mass affect interpretation
Impedance methods	Noninvasive estimation of IFV	Complexity and cost compared with other methods
	Provides insights into pulmonary fluid status	Conflicting study findings on efficacy

Each method has its strengths and weaknesses depending on the clinical scenario and specific goals of assessment in heart failure. Choosing the appropriate method often involves considering factors such as accessibility, cost, and reliability. ADHF, acute decompensated heart failure; BV, blood volume; BVA, blood volume analysis; CA125, cancer antigen 125; E, during echocardiography (E refers to the early blood flow into the left ventricle during passive relaxation, as visualized with pulse wave doppler); e', during echocardiography (e' refers to the movement of the septal mitral annulus during early left ventricular filling, as visualized with tissue doppler); HF, heart failure; IFV, intrathoracic fluid volume; IVC, inferior vena cava; LUS, lung ultrasound; NT-proBNP, N-terminal pro-brain natriuretic peptide; PCWP, pulmonary capillary wedge pressure.

### Plasma and Blood Biomarkers

The establishment of rational, quantitative, validated plasma biomarkers is being sought increasingly to diagnose, assess, and follow the progress of HF. Natriuretic peptides are released from the heart during increased stretch or stress of the atria or ventricles; levels of natriuretic peptides like BNP serve as indicators of cardiac stress. However, these biomarkers may not reliably predict clinical deterioration in mild congestive heart failure (CHF).^22^

Nonetheless, N-terminal pro-brain natriuretic peptide has shown utility as a nonspecific, but highly sensitive, diagnostic marker of acute decompensated HF (ADHF),^24,25^ but its effectiveness in guiding diuresis has yielded mixed results in terms of mortality, hospital readmission, and quality of life.^26–28^ While meta-analyses suggest potential benefits in patients younger than 75 years,^29,30^ N-terminal pro-brain natriuretic peptide is not a fully reliable index for guiding treatment decisions in ADHF.

Acute increases in Hb have long been used by nephrologists as a surrogate measure to guide fluid removal during hemodialysis, suggesting it could serve as a marker for effective plasma volume (PV) reduction and diuretic response in hospitalized HF patients. However, the timing of hemoconcentration is critical: late increases correlate with better outcomes, indicating effective therapy response, while early increases may signal excessive diuretic use and poorer outcomes.^5^ The preferred markers (Hb, Hct, or serum albumin) remain unclear because of influences by factors like subclinical bleeding and nutritional status. Additional methods are needed to guide decongestion therapy effectively.

Midregional proadrenomedullin shows promise as a prognostic marker in ADHF. It is a precursor fragment of adrenomedullin expressed in various tissues, including endothelial and vascular smooth muscle cells, where it helps stabilize endothelial barrier functions during volume overload stress^31^. Midregional proadrenomedullin has shown potential in predicting 90-day survival^32^ and rehospitalization^33^ in patients with HF. However, its release from different tissues in various conditions, such as interstitial lung disease,^34^ pneumonia,^35^ and sepsis,^36^ limits its specificity as a quantitative marker of volume overload in ADHF.

Serum creatinine (SCr) concentration is used often to guide fluid removal, but it reflects both renal glomerular filtration and muscle metabolism of creatine phosphate.^8,10^

Therefore, it can only be a reliable measurement of GFR during steady state creatinine production that do not hold for SCr in ADHF.^37^ While higher SCr levels are associated with worse outcomes in CHF, its use as an endpoint in ADHF trials is contentious.^38,39^

In the Diuretic Optimization Strategies Evaluation trial, an unexpected finding showed that a rise in SCr at 72 hours, typically considered adverse, was paradoxically linked to lower risks of death or HF events.^6^ This suggests that early declines in GFR might indicate a positive response to therapy. Nonetheless, SCr changes remain unreliable as end points for fluid removal therapy trials. Cystatin C, a protein reflecting GFR, is less affected by factors like muscle mass and diet compared with SCr. Estimating GFR using creatinine, cystatin C, or both is not validated in acutely ill patients.^10,40^

Carbohydrate antigen 125 is a transmembrane protein located in various epithelial tissues, functioning to protect epithelial surfaces from physical pressure.^41^ For unclear reasons, up to two-thirds of patients with ADHF can exhibit elevated carbohydrate antigen 125 levels.^42^ Cutoff values of <23 U/ml have shown to correlate with low risk of adverse events after discharge,^43^ and higher levels have been associated with increased risk of death and/or readmission.^44^ Studies have shown promise in its use to guide decongestive therapy,^45^ although this remains a nonspecific biomarker and must be used with caution in patients with complex comorbidities such as cirrhosis.

Neutrophil gelatinase-associated lipocalin (NGAL) is secreted into the urine within 3 hours of renal injury, reflecting severity and duration of insult.^46^ NGAL, unlike Cr, is not influenced by volume stressors. Biomarkers such as NGAL, kidney injury molecule -1, tissue inhibitor of metalloproteinase-1, and clusterin revealed that these were detectable after a brief period of renal ischemia, indicating their responsiveness to renal damage, yet none were expressed after near-fatal volume depletion despite a similar rise in SCr in both models.^47^ These biomarkers show promise in detecting renal damage, distinguishing it from hemodynamically mediated decreases in GFR.^37,47,48^ Analysis of the Reevaluation of Systemic Early Neuromuscular Blockade trial suggests that worsening renal function after aggressive diuresis in patients with HF can elevate SCr levels without renal tubular injury, indicating hemodynamic, rather than tubular, factors as the main contributors to SCr elevation in this scenario.^7^ A small subset of patients exhibited modest increases in the urinary biomarkers of tubular injury,^7,49^ but these did not correlate with worse outcomes, suggesting that overaggressive fluid management may compromise renal function due to hemodynamic changes rather than tubular injury.^7–10,50^ As such, acute SCr increases should be noted, but may not require a change in treatment strategy.

### Echocardiography

Echocardiography assesses ventricular size, function, and volume expansion. The PCWP can be estimated from the ratio of E/Ea or E/e′ using tissue Doppler measures of early mitral inflow velocity (E) related to the early diastolic flow velocity at the mitral annulus (Ea or e′). An E/e′ ratio >15 normally indicates a PCWP >15 mm Hg, when e' is the mean of the septal and lateral early diastolic flow velocities.^51^ However, the E/e′ ratio failed to predict the PCWP in one study of 106 patients with ADHF and left ventricular ejection fraction ≤30%^52^ and cannot predict PCWP in patients with significant cardiac valve disease where other Echo parameters may be required.^53^ Left atrial area is a supplementary sonographic variable that, when used in conjunction with E/e', can further increase sensitivity and specificity for predicting elevated PCWP.^54^

### Sonographic Assessments

Ultrasound provides many advantages to assess the functional status in ADHF. It is widely available, portable, safe, and cost-effective for monitoring patient progress and guiding therapy.

**Figure 2:**
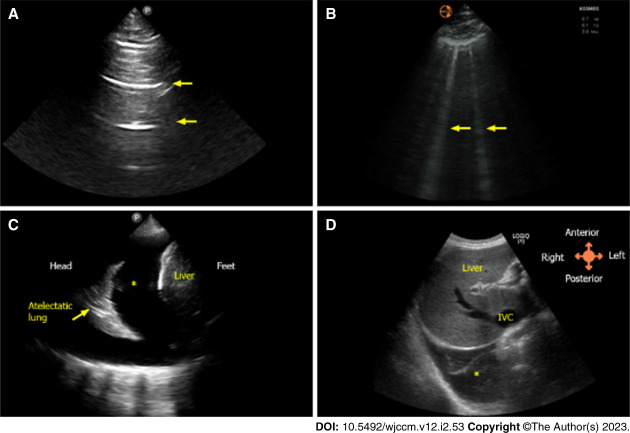
**Ultrasound images.** (A) Normal lung showing horizontal artifacts (A-line represented by arrows. (B) Vertical artifacts typically seen in congestion (B-line represented by arrows). (C) Pleural effusion indicated by asterisk. (D) IVC. IVC, inferior vena cava. Images used with permission.

LUS is particularly valuable for evaluating extravascular lung water content, with Kerley B lines as markers of pulmonary congestion.^55^ LUS demonstrates higher sensitivity for diagnosing cardiogenic pulmonary edema than plain radiographs and helps distinguish it from other causes of dyspnea.^56,57^ The Kerley B lines that are found on LUS are cleared during effective treatment of HF and correlate closely with clinical and laboratory markers of decongestion^58^ and can be effectively quantified by scanning a standardized number of anterolateral thoracic areas and assessing for the presence of ≥3 vertical lines (as shown in Figure 2). Therefore, they represent a quantitative, dynamic, reversible, and noninvasive direct method to assess pulmonary congestion. Nonetheless, the presence of B lines in conditions like acute respiratory distress syndrome reduces their specificity in patients with multiple comorbidities.

**Figure 3:**
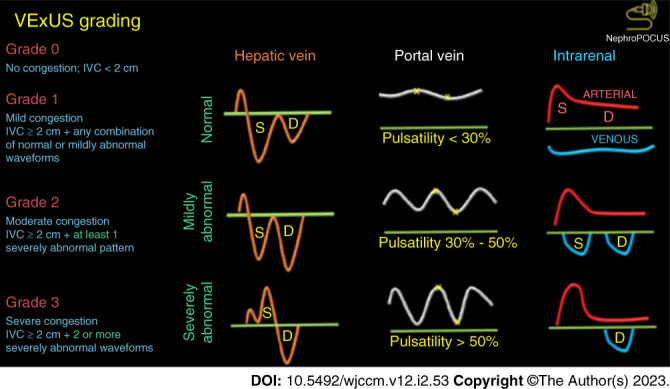
VExUS ultrasound depicting the relations between hepatic vein Doppler systolic (S) wave and diastolic (D) wave indicate varying degrees of congestion. Portal vein Doppler pulsatility similarly correlates with increasing congestion. Renal vein Doppler indicates increasing congestion when pulsatility becomes present in S and D waveforms. VExUS, venous excess ultrasound. Figure adapted from NephroPOCUS.com with permission.

Bedside ultrasound enables assessment of the inferior vena cava (IVC) collapse profile and diameter, often combined with LUS for better accuracy. The IVC can be visualized in its longitudinal axis from a subcostal position as it enters the right atrium, as illustrated in Figure 2. The results correlate closely with indices of congestion^59–61^ (Figure 3). IVC assessment can predict hospital readmission and visits to the emergency department post-ADHF treatment.^62,63^ However, evidence supporting its use in guiding decongestive therapy is lacking, and limitations exist in certain settings,^64^ such as positive pressure ventilation, severe right ventricle dysfunction, and tricuspid regurgitation. Nonetheless, both lung and IVC ultrasound offer accessible, cost-effective, and noninvasive means to evaluate central BV and pulmonary congestion in ADHF, warranting further investigation.

Venous excess ultrasound combines IVC ultrasound with Doppler measurements of flow rates in various veins.^65^ It grades congestion from zero to three, reflecting severity (Figure 3). It incorporates knowledge of changes to normal hepatic waveforms during the cardiac cycle (the A, S, and D waves) and their corresponding transmission to renal and portal venous systems under conditions of hypervolemia. Venous excess ultrasound correlates closely with right atrial pressure,^66^ although its role in guiding decongestive therapy is uncertain.^67^

## BV Analysis

The US Food and Drug Administration approved the BV analysis (BVA)-100 BV analyzer in 1998, which assesses BV by measuring the initial volume of distribution of labeled HSA ([^131^I]-HSA) after infusion. Blood samples are taken to gauge marker dilution, reflecting the fluid volume it is distributed in. While initially representing PV, labeled albumin disperses through capillaries over time, yielding a log-linear decline in plasma levels. Extrapolation to zero time determines the initial volume of distribution, estimating PV. BV is then calculated from PV and Hct. Although valuable, BV interpretation requires consideration of clinical context, comorbidities, hemodynamic values, and medications like diuretics or vasoactive drugs.

In addition, an initial BV measurement enhances the effectiveness of tracking volume status changes during treatment using Hgb or Hct. Peripheral Hct reliably indicates BV changes postinitial measurement, barring interventions like bleeding or transfusion. Hct sensors now allow continuous BV estimation during decongestive therapies, automatically pausing fluid removal if the change in Hct exceeds a predefined threshold (*e.g*., 5%–7%). Therapy resumes when Hct indicates adequate intravascular volume refilling. Subsequent BVA is needed if red cell mass changes occur, such as due to hemorrhage.^68–70^

Multiple studies have confirmed the validity of the BVA-100 as a quantitative measure of BV.^71–74^ In one study of 65 nonedematous ambulatory patients with HF, 43 (65%) were hypervolemic (mean deviation from normal values, +30 ±3%).^11^ Remarkably, the clinical assessment of hypervolemia was correct in only one half of these patients. This is important because increased BV has been linked to elevated PCWP and to a higher risk of death or urgent cardiac transplantation over a median follow-up of 719 days (*P* < 0.01).^11^ Consequently, clinically unrecognized hypervolemia is prevalent in nonedematous patients with HF, indicating more significant hemodynamic derangement and poorer outcomes. In one study, during admission for ADHF, 92% of patients exhibited hypervolemia based on BVA. Despite diuresis, total blood volume decreased only marginally from+39 to +30 ±16%, even with a decline in body weight of 6.9±5.2 kg and a net fluid loss of 8.4±5.2 L. Thus, some of fluid was lost from the interstitial compartment, accounting for 85%±15% of the total fluid loss.^75^ This example highlights the clinical insights gained from BV measurement during therapy. While pulmonary and peripheral edema signify excess interstitial fluid, assessing interstitial fluid volume (intrathoracic fluid volume [IFV]) is crucial. IFV can be deduced from the difference between extracellular fluid volume and PV.^76^ However, measuring extracellular fluid volume to calculate IFV in patients with HF is still experimental.

Diuretics primarily remove fluid from the interstitial compartment, often reflected in body weight loss, yet BV may remain expanded in patients with HF. Tailored therapy, including serial BV measures using BVA, is essential, especially for heart failure with preserved ejection fraction patients with diverse volume overload profiles.^77^ BVA-guided therapy has shown promise in reducing 30-day readmissions, as well as 30-day and 1-year mortality rates in patients with HF.^78^ Although constrained by a retrospective, nonrandomized design lacking precise treatment algorithms, this trial suggests that BV measurement could enhance clinical decision-making in ADHF. However, widespread adoption of BVA hinges on demonstrating its benefits in guiding ADHF treatment and conducting a cost-benefit analysis.

## Impedance Methods

The major symptom of ADHF is pulmonary congestion, making direct assessments of pulmonary fluid volume appealing.

Intrathoracic ICG quantifies CO and IFV noninvasively. The steady state of impedance provides measures of the electrical conductance of an applied current across the thorax. Because impedance is determined largely by conduction through an electrolyte containing fluid, it provides an indirect measurement of the extracellular fluid accumulated in the lungs and hence the IFV. Stroke volume, derived from impedance changes, allows calculation of Cardiac Index. ICG is performed by a Thoracic Bioimpedance Monitoring device with four dual sensors and eight leads on the chest and neck. Yu *et al.* reported the development of a progressive increase in the intrathoracic fluid content over 15 days before the appearance of pulmonary edema in patients developing severe HF.^79^ The intrathoracic fluid content correlated with the PCWP and with the fluid lost during treatment. In the BioImpedance CardioGraphy substudy of the Evaluation Study of Congestive Heart Failure and Pulmonary Artery Catheterization Effectiveness trial, ICG showed modest correlation with invasive CO and hemodynamics.^80^ ICG was reported to be superior to measurement of plasma BNP for assessing peripheral edema in CHF.^81^ However, its role remains uncertain because of conflicting study findings and its greater complexity and cost compared with LUS.

## Invasive Methods

A summary of invasive methods for assessing congestion is highlighted in Table 2.

**Table 2:** Invasive methods of evaluating congestion

Method	Pros	Cons
RHC	Direct measurement of cardiac filling pressures (*e.g*., PAWP) and PCWP, considered gold standard	Invasive procedure with associated risks (*e.g*., infection, bleeding)
	Essential in managing cardiogenic shock and guiding mechanical support decisions	Routine use for monitoring decongestion in ADHF not clearly justified by current evidence
	Provides precise hemodynamic data for treatment optimization	Effect on ADHF mortality remains inconclusive
Implantable devices	Allows continuous monitoring of PA pressure remotely (*e.g*., CardioMEMS)	Initial implantation procedure carries risks
	Demonstrated efficacy in reducing HF-related hospitalizations (*e.g*., 30% reduction in rehospitalization rates in CHAMPION trial)	Limited availability and high cost
	Early detection of worsening HF on the basis of pressure changes, enabling timely intervention	Long-term durability and reliability of devices need further study
	Additional monitoring capabilities (*e.g*., endotronix sensor for multiple vital parameters)	Limited data on mortality benefit
	Potential for integration with GDMT to improve outcomes (*e.g*., PROACTIVE-HF trial)	Specific devices may require different approaches and expertise for implantation and management
	Real-time monitoring of intravascular volume changes (*e.g*., FIRE1 IVC monitoring device)	Requires ongoing evaluation of safety and efficacy in clinical settings
	Meta-analyses support effectiveness in reducing rehospitalization rates	Implantable devices are not suitable for all patients because of procedural risks, cost, and individual medical needs

These methods offer varying levels of invasiveness in assessment of congestion in heart failure. Choosing the appropriate method depends on patient-specific factors, clinical goals, and available resources for monitoring and treatment. ADHF, acute decompensated heart failure; CHAMPION, CardioMEMS Heart Sensor Allows Monitoring of Pressure to Improve Outcomes in NYHA Class III Heart Failure Patients; GDMT, guideline-directed medical therapy; HF, heart failure; IVC, inferior vena cava; PA, pulmonary artery; PAWP, pulmonary arterial wedge pressure; PCWP, pulmonary capillary wedge pressure; RHC, right heart catheterization.

### RHC

RHC offers minimally invasive direct measurements of cardiac filling and pulmonary capillary pressures, traditionally considered gold standard for diagnosing and assessing ADHF response to treatment. While a multicenter randomized controlled trial reported superiority of invasive measurement of hemodynamics by RHC to clinical assessment for monitoring of decongestion,^82^ its effect on ADHF mortality remains inconclusive.^83^ Nonetheless, RHC proves beneficial in cardiogenic shock, guiding management decisions and the need for mechanical support.^84^ Therefore, routine monitoring of decongestion therapy by RHC in patients with ADHF is not clearly justified by the evidence available presently, but its utility in cardiogenic shock warrants ongoing evaluation.

### Implantable Devices

While many of the methods offer valuable insights into cardiac performance and congestion, they are impractical for us in patient's home. This is a notable limitation given evidence of gradual fluid retention leading to ADHF onset before noticeable weight gain. Implantable devices address this gap; for instance, the CardioMEMS heart sensor/transmitter records PA pressure continuously at home, allowing remote adjustments to medications. In the CardioMEMS Heart Sensor Allows Monitoring of Pressure to Improve Outcomes in NYHA Class III Heart Failure Patients trial, patients using PA monitoring had a 30% reduction in rehospitalization over 6 months, demonstrating improved outcomes. Meta-analyses further supported this method's efficacy in reducing rehospitalization rates.^85^ This pioneering trial showed enhanced outcomes with an implantable monitoring device, highlighting its effectiveness in predicting acute congestion episodes based on increases in left ventricular filling pressures. Meta-analyses further supported this method's efficacy in reducing rehospitalization rates.^86^

The Endotronix PA sensor, like CardioMEMS, offers hemodynamic monitoring and additional vital parameters such as BP, heart rate, weight, and oxygen saturations. It is currently being investigated in the PROACTIVE-HF IDE trial (ClinicalTrials.gov Identifier: NCT04089059), assessing whether adding PA pressure measurements to Guideline-Directed Medical Therapy and the cordella heart failure system can reduce HF-related hospitalizations or all-cause mortality over 6 months.

The FIRE1 IVC monitoring device, demonstrated in 2022, offers real-time measurement of IVC area changes in response to different volume states in sheep.^87,88^ Consisting of an implantable sensor in the IVC and an external unit communicating with a web application, its safety and potential to enhance HF outcomes in humans require further investigation.

## Conclusion and Future Directions

Congestion increases morbidity and mortality in patients with HF leading to frequent rehospitalizations. Current methods to assess congestion and determine optimal fluid status before discharge are limited. Natriuretic peptides are useful for diagnosis and prognosis in HF, but their ability to quantify fluid excess or guide therapy is limited by various confounding factors. Terms like SCr elevation, worsening renal function, and AKI are often used interchangeably, leading to premature discontinuation of decongestive therapies. While ultrasound offers simple methods for assessing volume status, further validation is needed. Direct measurement of BV with BVA is hindered by perceived complexity and limited studies on its predictive value. Bioelectrical impedance methods show promise but lack rigorous comparison with invasive hemodynamic data. Therapy guided by implanted hemodynamic monitors show potential for reducing mortality by detecting optimal rehospitalization times.

In an ideal scenario, comprehensive data on intravascular pressures, BVs, vascular characteristics, and fluid dynamics would inform personalized HF treatment. However, such information is currently lacking and challenging to interpret. Emerging technologies like handheld echocardiography and implantable hemodynamic monitors offer promise in advancing this field, potentially revolutionizing HF management.^85,89^
